# Nanocarrier drug delivery system: promising platform for targeted depression therapy

**DOI:** 10.3389/fphar.2024.1435133

**Published:** 2024-07-25

**Authors:** Xiaoying Feng, Ping Jia, Dingding Zhang

**Affiliations:** ^1^ College of Medical Technology, Chengdu University of Traditional Chinese Medicine, Chengdu, China; ^2^ Department of Neurosurgery Nursing, Sichuan Provincial People’s Hospital, University of Electronic Science and Technology of China, Chengdu, China; ^3^ Sichuan Provincial Key Laboratory for Genetic Disease, Sichuan Provincial People’s Hospital, University of Electronic Science and Technology of China, Chengdu, China

**Keywords:** depression, drug delivery system, nanocarriers, blood-brain barrier, antidepressants

## Abstract

Depression is a chronic mental disorder characterized by persistent low mood and loss of interest. Treatments for depression are varied but may not be sufficient cure. Drug-based treatment regimens have drawbacks such as slow onset of action, low bioavailability, and drug side effects. Nanocarrier Drug Delivery Systems (NDDS) has received increasing attention for brain drug delivery since it assists the drug through the blood-brain barrier and improves bioavailability, which may be beneficial for treating depression. Due to the particle size and physicochemical properties of nanocarriers, it presents a promise to improve the stability and solubility of antidepressants, thereby enhancing the drug concentration. Moreover, ligand-modified nanocarriers can be taken as a target direct medicines release system and reduce drug side effects. The purpose of the present review is to provide an up-to-date understanding of the Nanocarrier drug delivery system and relevant antidepressants in different routes of ingestion, to lay a foundation for the treatment of patients with depression.

## 1 Introduction

Depression is a common psychosomatic disorder that features chronic emotional sadness and lack of energy. Unlike ordinary emotional sadness, depression is defined by persistent low mood and absence of pleasure for at least 2 weeks clinically, accompanied by typical symptoms such as a change in appetite, insomnia or drowsiness, low self-esteem, and difficulty concentrating (Malhi and Mann, 2018; McCarron et al., 2021). The above symptoms lasting for 4–6 months are considered significant depression, and someone even has suicidal tendencies (Marwaha et al., 2023). World Health Organization statistics in 2023 reported that 280 million people worldwide were suffering from depression, and the number of people with depression increased even more dramatically during COVID-19 (Woody et al., 2017; Tang et al., 2024).

The causes of depression are related to a variety of factors, including family genetics, neurotransmitter imbalances, environmental stressors, and individual personality traits. Treatment options for depression involve the use of antidepressants, psychological counseling, and physical therapy. Although psychological interventions and social support are essential, medication is necessary for moderate or severe depression (Davidson, 2010). To prevent recurrent episodes of depression, most patients need to take antidepressants regularly for 6–12 months and even two consecutive years in patients over 50 years of age. However, some currently approved antidepressant drugs offer various side effects like restricted penetration, high toxicity, and low bioavailability (Mutingwende et al., 2021; Patel et al., 2022), and medications are ineffective in 1/3 of patients with major depression (Tao et al., 2024). Furthermore, Some antidepressants are susceptible to destruction by gastrointestinal enzymes and may not reach the site of action intact, resulting in reduced antidepressant efficacy (Buckman et al., 2018; Mutingwende et al., 2021; Monroe and Harkness, 2022). Nano-based drug delivery systems can be employed to overcome these limitations.

Nanocarriers drug delivery systems have been extensively developed to transport medicines through the blood-brain barrier (BBB) due to their various advantageous features (Sharma et al., 2019). Nanocarriers can improve the solubility of drugs to enhance their stability. Nanoformulations can be designed to release drugs under specific conditions, achieving sustained drug release, decreasing dosing frequency, and reducing toxic side effects (Agrawal et al., 2018; Sharma and Dang, 2020). Additionally, drugs loaded on nanocarriers are protected from enzymatic destruction, further enhancing the effect of antidepressants on the brain. This review targets common nanocarriers’ nature and function, considering the possible safety issues of NDDS and the challenges of clinical applications.

## 2 Strategies for drug delivery across the blood-brain barrier

The BBB is a selectively permeable physiological barrier between peripheral blood and brain tissue (Stamp et al., 2023). While useful for protecting the brain from harmful substances, it also limits drug entry. Depression is a neurological disorder of an imbalance of monoamine neurotransmitters, and several antidepressants have been developed based on this principle. How to get drugs across the BBB to the brain has been a focus of research in the treatment of depression. There are several strategies for delivering most types of drugs to the brain (Table 1). Either the drug is modified or encapsulated in a suitable carrier for transportation, or the BBB is modified to adapt it to drug passage. It is also possible to bypass the BBB to achieve this goal. In this regard, one can distinguish these four approaches in the following.

**TABLE 1:** Strategies for brain drug delivery.

Strategies	Instances	Benefits	Drawbacks	References
Nanoparticles	Nanoparticles for brain diagnostics or imaging	Cross the BBB by increasing the permeability under diseased states; Enhanced imaging	Dynamic change mechanisms of BBB are unclear	Dadfar et al. (2019)
	Other nanocarriers (e.g., lipid, gold, polymer, magnetic nanocarriers, etc.)	Active targeted delivery; Using specific physiological conditions for brain-targeted delivery	May cause neurotoxicity	Huang et al. (2019), Xiong et al. (2019), Zhao et al. (2022)
Viral vectors	Lentivirus; adenovirus	High efficiency of gene transfection	Safety concerns; May cause an immune response	Ewer et al. (2016), Shirley et al. (2020), Kamel et al. (2023)
Delivery across the permeable BBB in disease states	Inflammatory, traumatic, and degenerative diseases	Potentially cross the BBB	Dynamic changes of BBB and their underlying mechanisms remain unclear	Dong, (2018), Islam et al. (2020)
Exosomes	Isolated from NK cells, brain microvascular endothelial cells, etc.	Delivering genes across the BBB to brain and avoiding immune recognition	Difficult loading procedure; Necessitating exosome donor cells; Suboptimal pharmacokinetics	Zhang and Yu (2019), Kalluri and LeBleu (2020), Liang et al. (2021), Tenchov et al. (2022)
Changing the route of administration	Intranasal administration	Bypass the BBB for drug delivery	Suitable for low doses only	
Enhancing brain permeability	Borneol; Cereport	Temporarily open BBB	May be at risk of infection	Zhang et al. (2017)
Enhance brain drug uptake by non-invasive techniques	Focused ultrasound	Ability to increase BBB permeability and reduce efflux transporters	May lead to sub-lethal cellular damage or apoptosis	Pandit et al. (2020)
Delivery through active transporters in the BBB	Atp-binding cassette transporter; P-glycoprotein	Enabling efficient delivery of drugs to brain	Only suitable for small molecules	Azarmi et al. (2020)

### 2.1 Blood-brain barrier regulation

The BBB is mainly composed of brain microvascular endothelial cells and their intercellular tight junctions, intact basement membranes, pericytes, and astrocytes, of which the endothelium is the main structure. BBB permeability increases by opening tight junctions or inhibiting efflux systems to accommodate drug passage.

#### 2.1.1 Increasing the permeability of blood-brain barrier

Tight junctions are located between brain endothelial cells and are formed by ZO, Claudin, and Occludin proteins on endothelial cell membranes (Pandit et al., 2020). Tight junctions are a significant component in maintaining permeability and tissue homeostasis and constitute the physical barrier of the BBB (Wu et al., 2023). It can be artificially induced to open by interfering with the BBB through biological, chemical, and physical stimuli. Hypertonic solutions such as arabinose, mannitol, and urea infused into the internal carotid artery increase BBB permeability, enhance drug delivery, and return to normal osmolality in about 8 h (Sharma et al., 2023). Histamine, bradykinin, oleic acid, and other organisms or chemicals can also open the BBB briefly by injection. Moreover, the transportation of drugs to the brain can be modulated by physical disruption approaches based on optical, electrical, magnetic, and mechanical stimulation (Stamp et al., 2023). For example, focused ultrasound (FUS), a non-invasive technique that briefly opens the BBB in combination with microbubbles, has been used for brain delivery of antibodies, nano-agents, and growth factors (Wang et al., 2022). It is considered an effective way to diagnose and treat brain diseases.

#### 2.1.2 Overcoming efflux transporter activity

Efflux transport is the process by which various metabolites, toxic substances, neurotransmitters, and drugs are transported beyond the BBB through the efflux system. The efflux system consists of efflux transporters on the cell membranes or the BBB. They transport substances from the brain, eliminating wastes and preventing harmful substances enter. ATP-binding cassette (ABC) transporters, such as P-gp, BCRP, and MRP, are ubiquitously found in almost all organisms (Li et al., 2021). Thus, regulating the expression of efflux transporters on the brain microvascular endothelial cell membrane promotes the drugs across BBB.

P-gp is an important efflux protein that belongs to the ABC transporter family and is expressed in high abundance on the BBB. It regulates the uptake of endogenous and exogenous molecules and the clearance of waste products. Intentional modulation of P-gp activity has been studied for enhancing drug delivery to the brain and does show a bright prospect in animal models (Chai et al., 2022). Elkhayat et al. (2017) conducted a randomized double-blind trial recruiting drug-resistant epilepsy patients to compare the therapeutic effects of anti-epileptic drugs (AEDs) in combination with verapamil (P-gp inhibitor) and placebo. The results showed an improvement in the frequency of attacks and symptoms in patients taking verapamil. Verapamil increases the concentration of AEDs within the brain by inhibiting their efflux. Natural components derived from plants such as alkaloids, coumarins, flavonoids, and terpenoids have been found to inhibit ATPase activity and also serve as P-gp inhibitors (Mollazadeh et al., 2018).

However, there are certain research challenges associated with enhancing drug delivery through modulating efflux transporters. It is essential to know the corresponding efflux transporters for drugs and possess appropriate inhibitors, and typically more than one efflux system is involved in the drug efflux process (Banks, 2016).

### 2.2 Drug modification

Another way to enhance brain delivery of drugs may alter the physicochemical properties of a drug by modifying it, which in turn improves the transmembrane transport of the drug. Lipolysis may achieve the right lipophilicity for the drug to cross the BBB, and either insufficient or too much lipolysis will affect the penetration rate of the drug. In addition, prodrugs can be synthesized to overcome the low bioavailability of medications due to gastrointestinal digestion. A prodrug is a compound obtained by modification of a drug that is inactive or less active *in vitro* but is transformed *in vivo* to release activity and exert medicinal effects. Inatani et al. (2023) demonstrated TP0473292 as an ideal oral prodrug by animal studies. TP0473292 is an adamantane carboxylic acid ester prodrug, which can be used as a novel antidepressant that enhances metabotropic glutamate 2/3 receptor antagonist oral bioavailability.

### 2.3 Bypassing the BBB for drug delivery

In addition to the standard oral and IV administration of drugs, drugs can be delivered directly to the brain by bypassing the BBB. For example, intracerebroventricular injection administration allows the drug to go directly to the brain to work, but this is an invasive route that can cause intracranial infections. In addition, intranasal administration bypasses the BBB to reach the central nervous system (CNS) via the mucous membranes of the olfactory region, which is non-invasive and improves drug distribution in the brain. In 2019, esketamine intranasal formulation was approved by the FDA for treatment-resistant depression (Cristea and Naudet, 2019).

### 2.4 Drug delivery system

#### 2.4.1 Viral vectors

Viral vectors are tools that utilize a modified virus to deliver genetic material to a host. The virus carrying the target gene is delivered to the host cell to alter the host cell’s gene by utilizing the high efficiency of viral transfection. Common viral vectors have been widely used in vaccine development and gene therapy, including lentiviruses, adenoviruses, and adeno-associated viruses (AAV) (Ewer et al., 2016; Milone and O’Doherty, 2018). In recent years, viral vector-mediated genes have opened up more possibilities for treating neurological diseases. Viral vectors have shown rosy therapeutic efficacy in studies for treating Parkinson’s, Alzheimer’s disease, and brain tumors, and the lack of suitable animal prediction models and species differences have hindered the transition of viral vectors from the laboratory to clinical applications (Choudhury et al., 2017; Lundstrom, 2023). AAV9, the AAV serotype, has been extensively researched for its capacity to penetrate the BBB to transduce astrocytes (Liu et al., 2021). While gene therapy has demonstrated potential in animal and cell investigations for CNS diseases, the majority of clinical trials have yet to be successful enough. Finding new approaches to increase the efficacy of gene therapy for CNS disorders is still crucial (Liu et al., 2021).

Viral vector-based gene therapy medications have been approved for clinical, but more viral vectors are still in the preclinical and laboratory stages (Shirley et al., 2020). The obstacles to its development are mainly the possibility of causing host immune responses or genetic mutations, and the safety assessment of vectors with high replication capacity is even more important (Lundstrom, 2023). Currently, viral and non-viral vectors are the main methods for gene delivery. Non-viral vectors, such as liposomes, micelles, polymeric substances, and various nanoparticles, are frequently utilized (Mantz and Pannier, 2019). Nanocarriers increase drug loading and gene therapy efficacy and exhibit superior biosafety and targeting properties compared to viral vectors (Pan et al., 2021).

#### 2.4.2 Exosomes

Successive invaginations of the cell membrane form multivesicular vesicles fuse with the cell membrane and are released extracellularly to form exosomes (Pegtel and Gould, 2019). They contain many cellular components such as proteins, DNA, RNA, and cell surface proteins. They can transmit these signaling molecules to other cells, thus affecting the biological behaviors and functions of the recipient cells. Hence, exosomes are regarded as a new cell-to-cell information transfer system (Zhang and Yu, 2019; Kalluri and LeBleu, 2020).

Tavakolizadeh et al. (2018) indicated that miRNAs and exosomes were novel biomarkers for the diagnosis and treatment of depression. Li et al. (2020) demonstrated exosomes from NK cells carrying miRNA-270 alleviated mouse depressive symptoms. Fluorescently labeled exosomes were injected by tail vein, and fluorescent signals were detected in the brains of mice after 12–24 h. ELISA analysis showed a reduction in pro-inflammatory cytokines associated with depression. The results displayed that NK cell-derived exosomes alleviated depressive symptoms in mice by reducing depression-associated inflammatory factors (Li et al., 2020). Fan et al. (2022) also demonstrated that microglia-derived exosomes enriched in miR-146a-5p induced downregulation of factors that inhibit excitatory neurons (predominantly KLF4) and alleviated the depressant drug phenotype in a rat model of depression.

Exosomes are natural products secreted by cells and are, therefore, biocompatible and have low immunogenicity. However, there are still many challenges to the clinical application of exosomes as drug-delivery vehicles. It is difficult to produce and purify, and their drug-carrying capacity may be limited, restricting the large-scale application of exosomes in the clinic (Mondal et al., 2023).

#### 2.4.3 Nano drug delivery system

Unlike viral vectors and exosomes, nanocarriers are synthetic materials with significant drug delivery advantages and unique physicochemical properties. The major advantage of NDDS is its ability to increase the bioavailability of drugs (Khizar et al., 2023). Nanocarriers protect drug molecules from enzymatic damage and ensure their intact entry into the bloodstream, thus improving drug stability (Liu et al., 2023). Moreover, control over the drug release rate can be obtained by modifying the composition and architecture of the nanocarriers. It prolongs the antidepressants’ beneficial effects on the body, hence lowering the frequency of their administration. Nanocarrier systems can also be designed to enhance targeted delivery by designing specific surface antibodies or ligands (Miao et al., 2023). This means that antidepressants can act directly on particular lesions in the brain, increasing efficacy while reducing toxicity to normal tissue.

## 3 Nanocarriers in the treatment of depression

Currently, traditional formulations used to treat depression have some drawbacks such as limited penetration, frequent dosing, and side effects. It is a barrier to antidepressants reaching the brain through traditional formulations. Nano-formulations are expected to overcome this limitations and might be an effective treatment for depression (Table 2).

**TABLE 2:** Comparison of traditional formulation and nano-based formulation.

Treatment	Pros	Cons	References
Nano-based formulations	Control the release of the drug and prolongs its circulation time in the bloodstream, thereby increasing bioavailability; Ligand complexed nanocarriers improve drug specificity; Reduce the frequency and dose of administration, thereby reducing side effects; Can be used in oral, intranasal, and parenteral routes of administration	High cost and the need for special equipment and techniques may increase the financial burden of treatment; The safety of long-term use needs to be further studied and evaluated; Still in the research phase, more clinical studies needed.	Zorkina et al. (2020), Song et al. (2023), Unnisa et al. (2023)
Conventional formulations	More economical and convenient; The technologies are mature; Widely applied in clinical; Remain the first choice for most patients	Low bioavailability, delayed therapeutic efficacy, adverse side effects, and high dose-induced drug toxicity; Weak permeability, does not easily cross the BBB; Antidepressants are ineffective in some patients	Mutingwende et al. (2021), Patel et al. (2022)

### 3.1 Targeting strategies of nanocarriers

A suitable CNS drug delivery system should have the capability to target the BBB and reach the therapeutic area. The Enhanced Permeation and Retention (EPR) effect serves as a fundamental mechanism for nanocarrier targeting strategies. The EPR effect refers to the fact that during tumor growth, the vascular structure of the tumor region is more sparse compared to normal tissue, leading to increased permeability of the tissue (Cheng et al., 2021). This pathological change is considered beneficial as it enhances the accumulation of nanoparticles within the tumor area, leading to the formation of elevated concentrations. However, there are some unpredictable elements to this passive targeting (Furtado et al., 2018). If the vasculature is not sufficiently sparse, the nanocarriers may struggle to accumulate effectively at the desired site. Additionally, this delivery modality is also affected by multiple factors such as high intertumoral fluid pressure, poor blood flow, and other factors.

Coupling specific targeting molecules on the surface of nanocarriers, these ligands bind to specific receptors or markers on the surface of diseased tissues or cells to obtain better specificity (Cheng et al., 2021). Research has demonstrated that the p11 gene is involved in the modulation of brain serotonin levels and has been studied for applications in gene therapy for depression (Gałecka et al., 2022). Brain microvascular endothelium is rich in insulin-like growth factor II (IGF-II) receptors, and Gandhi et al. utilized IGF-II monoclonal antibodies coupled to liposomes to enhance targeted delivery to the p11 gene (Gandhi et al., 2019). The analysis of SD-PAGE and Caumas Brilliant Blue staining indicated that IGF-II bound to the surface of liposomes well. They utilized fluorescence-emitting plasmid DNA instead of p11 cDNA to visualize the localization of the targeting agent within rat brain tissue. This indicated that the specific formulation displayed increased fluorescence and uptake of the formulation at every stage in comparison to the non-targeted formulation (Gandhi et al., 2019). IGF-II coupled with liposomal may be considered a novel gene therapy for depression. Ge et al. prepared ketamine (KA) loaded nanopreparations with the bifunctional peptide (Ang-2-Con-G, AC) as a targeting factor (Ge et al., 2023). A peptide (Ang-2) specifically binds to relevant proteins in the BBB to improve penetration of the BBB. Another peptide (Con-G) is directed towards the transport of KA to hippocampal and prefrontal cortical areas. Morris water maze test and the Novel Object Recognition test showed that KA could be directed to accumulate in the treated area, resulting in the recovery of cognitive functions.

Ligand-modified nanoparticles are widely explored for brain delivery. Furthermore, the characteristics of nanomaterials, such as size, shape, chemical composition, surface charge, and other factors, also play a crucial role in the overall efficacy of nanocarrier. Through purposeful design, nanomaterials may transport drugs to the target site efficiently (Table 3) (Cheng et al., 2021; Li et al., 2021).

**TABLE 3:** A summary of implications of parameter design of nanocarriers for achieving brain-targeted delivery.

Particle parameters	Examples	Impacts of parameters on BBB	References
Size	<100 nm	The BBB is less permeable to macromolecules, and the nanocarrier particle size between 50 and 100 nm is optimal. The particle size directly affects the efficiency of BBB penetration and clearance from body	Nowak et al. (2020)
Shape	Spherical, tubular, rod-shaped, sheet-like, irregularly shaped	Generally, spherical or spherical-like nanocarriers are more likely to cross the BBB due to their lower resistance in blood flow. However, studies have shown that rod-shaped nanoparticles with high aspect ratios increased the adhesion capacity resulting in longer circulation time, and crosse BBB more efficiently	Song et al. (2023)
Surface charge	Low (−1 to −15 mV) to moderate (−15 to −45 mV) negative Zeta potential	High Zeta potential may alter the integrity and permeability of BBB, cause cerebral endothelial toxicity. Neutral and negative potentials provide a safer structure and longer lifetime	Song et al. (2023)
Ligands	Antibodies	OX26mAb, 19B8mAb etc. targeted transferrin. IGF-II mAb increases p11 protein levels and may improve depression	Gandhi et al. (2019)
	Proteins	Transferrin targets transferrin receptors in brain capillary endothelial cells. Lactoferrin receptors are found in the brain, including the microvascular system and neurons. Low-density lipoprotein receptor-related protein (LRP)	Naidu et al. (2023), Teixeira et al. (2023)
	Peptides	Apolipoprotein E can target LRP. Cell penetrating peptides, peptide B6, THR, etc. can be used to target transferrin	Gardner and Levin (2015)
	Surfactants	Polysorbate 80, polysorbate 20, poloxamer 188, etc. can target low-density lipoprotein receptors	Zakharova et al. (2019), Dri et al. (2021)
	Others	Folic acid, glutathione, leptin, thiamine, etc.	Jain and Jain (2015), Teixeira et al. (2023)

### 3.2 Types of nanocarriers

**FIGURE 1:**
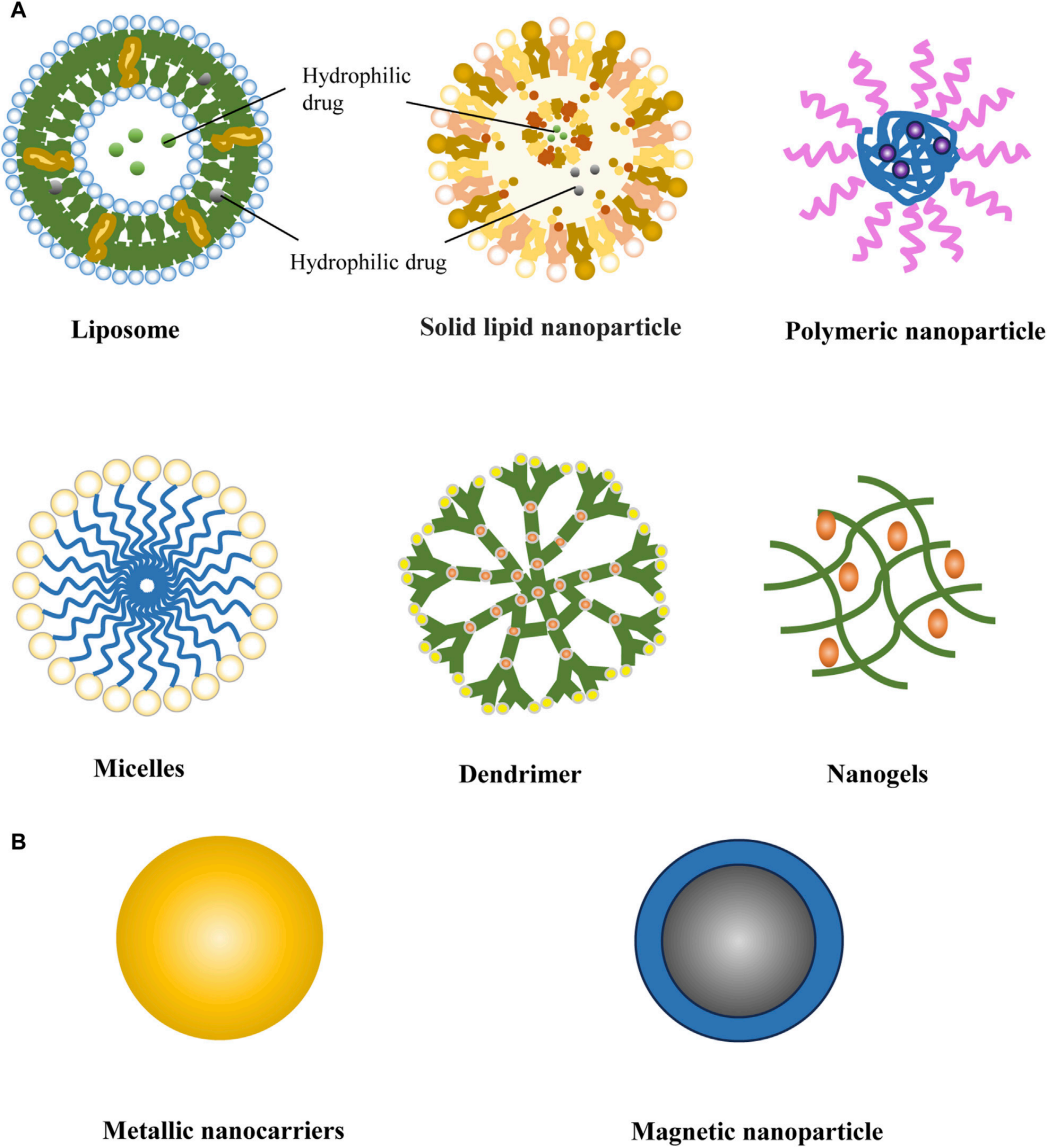
Schematic representation of various types of nanocarriers used commonly for nano-drug delivery systems. **(A)** Organic nanocarriers: liposom, solid lipid nanoparticle, polymeric nanoparticle, micelles, dendrimer, nanogels; **(B)** Inorganic nanoparticle carrier: metallic nanocarriers and magnetic nanoparticle.

Nanocarriers are mainly classified into organic nanocarriers and inorganic nanocarriers (Patel et al., 2022). Organic nanocarriers typically consist of polymers, lipid materials, proteins, and other organic substances, such as liposomes, nylon, nanocapsules, and micelles. Inorganic nanocarriers are composed of inorganic materials like metal oxides, and magnetic nanomaterials etc. (Figure 1). The nanocarriers were categorized into polymer-based carriers, lipid-based carriers, and metallic carriers according to the constituent materials, which are applied to the study of antidepressant therapy.

#### 3.2.1 Lipid-based carriers

##### 3.2.1.1 Liposome

Liposomes are spherical drug delivery vehicles ranging from 25–1,000 nm in diameter, composed primarily of natural or synthetic phospholipids (Li et al., 2019). In the aqueous environment, the hydrophilic head is exposed to the inner and outer aqueous phases, and the hydrophobic tail aggregates to form closed vesicles with a stable bilayer structure (Has and Sunthar, 2020). Liposomes’ unique structure and composition determine that they can encapsulate hydrophilic drugs and transport lipophilic drugs (Fan et al., 2021; Guimarães et al., 2021). Liposomes have been used as drug carriers in therapeutic studies for malaria, cancer, breast cancer, and other diseases due to their targeting, controlled release, decreased drug toxicity, and enhanced drug stability (Almeida et al., 2020; Memvanga and Nkanga, 2021; Forouhari et al., 2022).

Dandekar et al. (2018) loaded xenon (Xe) into liposomes for mouse models of drug-administered depression to prove the antidepressant effects of Xe. The stimulatory effects of Xe-liposomes on locomotion in mice were conducted by spontaneous locomotion monitoring and forced swimming tests in rats. Behavioral studies in the mouse models of depression have shown that rats spend less time swimming and standing still and are much more active. Additionally, a group led by Nodari prepared liposomes loaded with Vortioxetine hydrobromide (VXT-Ls) to improve the stability of the refractory antidepressant Vortioxetine hydrobromide (VXT) stability and reduce drug-free toxicity (Nodari et al., 2022). This showed that the cell survival rate in the VXT-Ls group was significantly higher than in the VXT group at the same drug concentration. Studies have indicated liposomes with amphiphilic properties are positive carriers for transporting hydrophilic and hydrophobic drugs.

The transition of liposomes from the laboratory to large-scale production is a complex process in which much work remains. The stability of liposomes is a crucial factor in the safe transportation of encapsulated drugs *in vivo* to exert their therapeutic effects and a significant challenge in the production process of liposomal formulations (Guimarães et al., 2021). Therefore, there is also a need for more rapid assays to provide safety assurance for liposome characterization and quality (Shah et al., 2020).

##### 3.2.1.2 Solid lipid nanoparticles

Solid lipid nanoparticles (SLN) are discs or elliptical spherical particles composed of solid lipids, water, and surfactants at room temperature, and the drug may be attached to the surface of the carrier or encapsulated in a core (Scioli Montoto et al., 2020). SLN has the advantages of enhanced drug penetration, targeted delivery, loading of amphiphilic drugs, and protection from hepatic clearance for safe transportation and improved drug efficacy (Mishra et al., 2018). SLN prepared by various techniques (e.g., cold or hot homogenization, microemulsion method, ultrasound or high-speed homogenization, etc.) has been widely investigated for applications in vaccines, anticancer, antimicrobial, and gene delivery (Rajpoot, 2019).

Khan et al. (2021) demonstrated the antidepressant effects of diosgenin in a mouse animal model. Its low water solubility and low bioavailability affect its efficacy as an antidepressant. Diosgenin-loaded SLN was used to assess its antidepressant potential through a variety of behavioral tests, and the results indicated that SLN-encapsulated diosgenin had a positive effect in alleviating depressive behaviors. Thus, SLN allows improved bioavailability and penetration of drugs. The tricyclic antidepressant Amoxapine (AMX) is difficult to dissolve in water as well as to overcome first-pass metabolism, which affects its use as an antidepressant. Pawar et al. (2023) prepared solid liposome-encapsulated amoxapine (AMX-SLNs) by using a single emulsification method to enhance its antidepressant effect. Plasma and brain tissue AMX concentrations were determined using high-performance liquid chromatography (HPLC) and LC-MS/MS methods. Results confirmed a 1.6-fold increase in bioavailability and a 5.8-fold increase in brain drug concentration of AMX-SLN compared to oral AMX. Results indicated that SLN is an effective transport vehicle for increasing the distribution concentration of insoluble drugs in the brain.

##### 3.2.1.3 Nanostructured lipid carriers

Due to the instability of SLN, possible aggregation during storage, and limited drug loading capacity, nanostructured lipid carriers were developed to overcome these problems. Nanostructured lipid carriers (NLC) are regarded as an upgraded version of SLNs, containing more solid lipids than compared to liquid lipids, allowing for increased capacity for drug incorporation. Compared with SLN, NLC has better stability, controlled-release capability, and higher drug-loading capacity (Viegas et al., 2023).

Gul et al. (2022) designed Agomelatine-loaded nanostructured lipid carriers (AGM-NLC) to study the antidepressant effect of Agomelatine (AGM), a novel antidepressant encapsulated by NLC. The drug’s ability to act as an antidepressant was evaluated by forced swimming and tail-hanging tests, revealing that mice in the AGM-NLC group exhibited significantly decreased resting times compared to the control group. The pathogenesis of depression is linked to neurological impairment and inflammation. Therefore, they also evaluated the damage of AGM-NLC on brain neurons in mice, focusing on the effects of the drug on the levels of the inflammatory mediators TNF-α and COX-2. The frontal cortex of the mouse brain was examined under a microscope using Ni-staining, and AGM-NLC effectively maintained the quantity and morphological traits of the remaining neurons. Moreover, immunohistochemistry revealed decreased levels of TNF-α and COX-2 expression (Gul et al., 2022). These findings indicated that the administration of AGM may decrease *in vivo* toxicity and enhance the antidepressant properties when combined with NLC.

#### 3.2.2 Polymer-based carriers

##### 3.2.2.1 Dendrimer

Dendritic macromolecules are specialized circular structures with many potential active sites and hyperbranched interactions (Shcharbin et al., 2017). Drugs could be encapsulated in the interior of dendritic macromolecules or affixed to their surfaces by hydrophobic forces or covalent binding, depending on the stability of the drug molecule (Dening et al., 2016; Patel et al., 2022). The unique structure and surface functional groups of dendritic macromolecules provide benefits for drug delivery, including biocompatibility, prolonged circulation time, and targeted delivery (Mutingwende et al., 2021). Polyamide-amine (PAMAM) is a tree-like macromolecule widely utilized in biomedical fields (Wilczewska et al., 2012). Tripathi et al. addressed the limitations associated with curcumin, including its instability, high metabolism, and poor absorption, by developing a novel delivery platform utilizing generation 4 poly-amido-amine (G4 PAMAM) as a carrier to enhance the bioavailability of curcumin (Tripathi et al., 2020). They conducted force swimming and tail-hanging tests with a stress-induced mouse model. Plasma curcumin levels were analyzed by reverse high-performance liquid chromatography to assess the pharmacokinetic parameters *in vivo*. The results showed an increase in peak concentration (Cmax), a delay in reaching maximum concentration, and a decrease in the mouse resting time in water. This suggests that G4 PAMAM exhibits a slow-release property that enhances efficacy and diminishes curcumin metabolism (Tripathi et al., 2020).

##### 3.2.2.2 Polymer micelles

Polymeric micelles are nanostructures composed of amphiphilic block polymers (Nishiyama and Kataoka, 2006). When the surfactant concentration reaches the critical micelle concentration, the amphiphilic molecular polymer self-assembles in aqueous solution to form an ordered arrangement of thermodynamically stable lipophilic core and hydrophilic outer shell (Owen et al., 2012; Cagel et al., 2017). Polymeric micelles are known for their small size, structural stability, and minimal toxicity, and they also regulate the release of hydrophobic drugs and enhance drug availability and duration in the body (Kataoka et al., 2001; Rub et al., 2013). The highly functional core and shell allow them to play a vital role in drug delivery systems. There is an interaction between the hydrophobic nucleus and the drug, which enables the drug to be retained in the lipophilic nucleus, controlling the slow release of the drug from the micelle (Lin et al., 2003; Zhang and Zhuo, 2005; Hwang et al., 2020). Polymer micelles typically have a particle size ranging from 10 to 200 nm, making them difficult to recognize and capture by the endothelial reticular system in blood circulation. This characteristic enables the drug-loaded polymer micelles to remain stable and extend the time circulating in the bloodstream (Croy and Kwon, 2006; Lu and Park, 2013). The hydrophilic shell reduces the binding of serum proteins to the complement system and avoids the loss of the encapsulated drug during the body’s circulation (Xiao et al., 2011). Therefore, hydrophilic blocks should be added to the design of polymer micelles to avoid polymer adsorption by plasma proteins or removal from the body by complement activation (Hwang et al., 2020). Polyethylene glycol has been the most commonly used hydrophilic polymer in polymer micelles. It also showed that polyethylene glycol is permeable to mucus, which helps nanomedicines to cross the mucosal barrier effectively (Maisel et al., 2016).

Abourehab et al. (2018) analyzed the distribution concentration of polymer micelles loaded with dapoxetine (DPX-PM) in the brain. Results showed that DPX-PM reached its maximum drug concentration in the brain after 1.5 h of oral administration, and the drug concentration was 1.2 times that of ordinary DPX tablets. This illustrates that polymeric micelles may not only overcome the aggressive physical environment of the gastrointestinal tract but also prolong the duration of the drug’s residence in the brain (Abourehab et al., 2018). Moreover, El-Helaly and Rashad (2024) investigated the antidepressant activity of Mirtazapine (MTZ) through *in vitro* solubility and coefficient of dissolution studies, as well as drug content determination *in vivo*. The non-ionic nature of polymeric micelles reduces electrostatic repulsion between drug molecules, forming a hydrophilic outer shell around the nucleus. It greatly improves the solubility and stability of drug-loaded micelles. This finding indicates that polymeric micelles could enhance the oral bioavailability and solubility of drugs like MTZ (El-Helaly and Rashad, 2024).

##### 3.2.2.3 Poly(lactic-co-glycolic acid)

Poly(lactic-co-glycolic acid) (PLGA) is a copolymer formed by the random polymerization of lactic acid and hydroxyacetic acid (Furtado et al., 2018). Parenteral administration of PLGA decreases the frequency of dosing, improves patient compliance, reduces dosage, and mitigates systemic side effects (Su et al., 2021). The FDA has approved PLGA for use as a parenteral transport vehicle in drug delivery systems (Singh et al., 2021).

Cayero-Otero et al. (2019) employed venlafaxine (VLF) as an antidepressant drug model encapsulated within PLGA nanomaterials (VLF-PLGA NPs) to achieve controlled release of VLF, thereby augmenting therapeutic levels of venlafaxine in the bloodstream. *In vivo*, biodistribution studies demonstrated that PLGA could cross the BBB to deliver the drug to the brain to improve bioavailability. Duloxetine hydrochloride (DXH) is a psychotropic drug for the treatment of major depressive disorder. Its poor water solubility and extensive metabolism necessitate frequent dosing to compensate for reduced bioavailability. Singh et al. prepared DLX-loaded PLGA (DLX-PLGA) using an emulsion solvent evaporation technique to enhance its antidepressant activity (Singh et al., 2021). They employed HPLC to analyze DLX levels present in blood and brain specimens, revealing that concentrations were higher than those of commercially available drugs in blood and brain. Moreover, the forced swimming test showed that the administration of DLX-PLGA notably reduced the immobility duration of the mice. Studies have shown that intramuscular injection of DLX-loaded polymer nanoparticles is a viable strategy to reduce the frequency of dosing and may help promote patient compliance for the effective management of major depressive disorder.

##### 3.2.2.4 Chitosan

Chitosan is a biopolymer with good biocompatibility and biodegradability (Silant’ev et al., 2023). Its degradation components are non-toxic, do not re-accumulate within the body, and are non-antigenic, increasing its biomedical utility in the CNS due to its potential ability to cross the BBB (Silant’ev et al., 2023).

Tong et al. (2017) prepared PLGA-chitosan nanoparticles loaded with desvenlafaxine (DVF PLGA-CN) through solvent-emulsion evaporation technique and evaluated the antidepressant effectiveness of desvenlafaxine in a male rat depression model. DVF PLGA-CN nanoparticles significantly enhanced the levels of serotonin, norepinephrine, and dopamine within the brain when compared with depressed controls. Biochemical results showed that DVF PLGA-CN nanoparticles effectively alleviated depressive symptoms in rats. Salem et al. (2023) prepared and optimized DXH-loaded PLGA-chitosan nanoparticles (DXH-PLGA-CS-NPs) to improve the bioavailability of DXH. The sucrose preference test and forced swimming test indicated increased mobility and sucrose consumption in rats after the administration of DXH-PLGA-CS-NPs. This suggests that DXH-PLGA-CS-NPs may be a safe, stable, and efficient method for delivering drugs to address depression.

In another study, Lima et al. (2019) evaluated changes in Swiss mice exposed to different concentrations of chitosan-coated zein nanoparticles (ZNP-CS) for a short period. They assessed cognitive and behavioral changes in the mice through experiments such as behavioral tests like mazes and target recognition and motor performance assessments. Results showed that mice were sedentary for a longer time in the hanging tail test, that ZNP-CS induced depressive-like behaviors, and that exposure to ZNP-CS may lead to neurotoxic effects. This lays the foundation for the biosafety assessment of chitosan in the field of drug delivery.

#### 3.2.3 Metallic nanocarriers

Metallic nanoparticles have the advantages of the therapeutic efficacy of drugs by enabling targeted delivery and mitigating multidrug resistance. They are widely employed as vehicles for delivering a range of therapeutic agents such as antibodies, nucleic acids, chemotherapeutic drugs, etc. (Chandrakala et al., 2022). Metallic nanocarriers enhance the aqueous solubility of hydrophobic pharmaceutical compounds, prolong the systemic circulation of drugs in the bloodstream, and impede or prevent the rapid renal excretion of drugs (Chandrakala et al., 2022). In the field of drug delivery, common metal nanocarriers are gold, silver, iron oxide, and zinc oxide nanoparticles (Rathnam et al., 2024).

Saeidienik et al. (2018) studied the effect of iron oxide nanoparticles on lipopolysaccharide-induced depressive behavior in rats. Rats were exposed to the forced swim test (FST) and the open field test (OFT), with groups receiving iron nanoparticle treatment showing an increase in swimming duration and a decrease in the duration of immobilization. Results indicated the beneficial affect of administering iron nanoparticles in reducing depressive symptoms, which may be attributed to the influence of iron oxide nanoparticles on neurotransmitters and inflammatory factors associated with depression. Khadrawy et al. (2021) investigated the antidepressant effects of curcumin-coated iron oxide nanoparticles (Cur-IONs) using a rat model of rifampicin-induced depression. Lipid peroxidation, nitric oxide levels, monoamine oxidase activity, and monoamine neurotransmitters were assessed in the brain tissue of rats. Results showed that Cur-IONs may modulate depressive symptoms by increasing the synthesis of monoamine neurotransmitters and preventing their degradation.

Similarly, Fahmy et al. (2024) augmented the therapeutic efficacy for depression by synergistically incorporating curcumin with zinc oxide nanoparticles, leading to improved pharmacokinetic properties of curcumin. The study reported that exposure to zinc oxide nanoparticles (ZnO NPs) may reduce monoamine neurotransmitters in the brain (Jeyhoonabadi et al., 2022). Jeyhoonabadi et al. (2022) identified the protective impact of the novel psychotherapeutic medication betaine against ZnO NPs-induced toxicity in mice. Behavioral tests such as the FST showed that betaine mitigated depression-like behavior in mice. Furthermore, the histopathological in the hippocampus of mice indicated that the majority of cells in the group treated with betaine and ZnO NPs exhibited preserved morphology and dimensions.

## 4 Antidepressant medications

Antidepressants currently approved for marketing by the FDA to alleviate depressive symptoms by modulating monoamine neurotransmitters and are mainly categorized as follows (Table 4).

**TABLE 4:** Summary of common antidepressants.

Antidepressant	Instance	Mechanism	Disadvantage	Ref.
MAOIs	Selegiline Tranylcypromine	Increases brain intercellular neurotransmitter concentrations by inhibiting the clearance of NE, 5-HT, and DA	Combining it with several drugs have serious toxic side effects and is gradually being replaced.	Ricken et al. (2017), Tábi et al. (2020)
TCAs	Imipramine Amitriptyline	Blocking reuptake of NA and 5-HT by NAergicand 5-HTergic nerve endings increases the concentration of synaptic gap monoamine transmitters	Greater anticholinergic and cardiovascular adverse effects, more contraindications and drug-drug interactions, and a narrower range of safety limit its clinical use	Sanganahalli et al. (2000), Ding et al. (2016), McClure and Daniels (2021)
SSRIs	Fluoxetine Sertraline Paroxetine	Selective inhibition of 5-HT recycling indirectly elevates synaptic gap 5-HT concentration	Side effects such as increased bleeding rate, hyponatremia, hepatotoxicity, and sexual dysfunction	Rothmore (2020), Jannini et al. (2022), Kalbouneh et al. (2022)
SNRIs	Venlafaxine Duloxetine milnacipran	Blocking the reuptake of the neurotransmitters serotonin and NE in the brain	It is the first-line clinical antidepressant, but more than half of depressed patients still fail to achieve a cure in the first round of treatment	Furukawa et al. (2019), Rothmore (2020)

Nearly half of the patients with significant depression still do not respond to first-line antidepressants. Also, there are side effects of conventional antidepressants and interactions between drugs or drugs and food, which has prompted scientists to start exploring more antidepressants. Several natural, plant-derived active ingredients are beginning to be studied for the treatment of depression, which may have fewer side effects and show better biocompatibility than synthetic drugs.

Many natural ingredients apply to antidepressant research, such as icariin, ginsenoside, chrysin, etc. However, its ability to cross the BBB is limited, and efficacy through oral delivery is inadequate. Li et al. (2022) explored the antidepressant mechanism of ginsenoside Rg1, confirming that ginsenoside Rg1 down-regulates the expression of GAS5 to attenuate microglia activation and alleviate depressive-like behavior. Yu et al. (2023) designed a drug-carrying platform consisting of Borneol and graphene to wrap ginsenoside Rg1 (BO-GO-Rg1). Behavioral tests in rats utilizing Borneol (BO) to enhance BBB permeability demonstrated that the compound BO-GO-Rg1 alleviated symptoms of anxiety and depression in rats 1-week following administration of BO-GO-Rg1 (Yu et al., 2023). Moreover, C-glycosylated flavonoids have been found to have antidepressant activity in the passion flower leaves, which grow mainly in Brazil. Alves et al. (2020) prepared nanoparticle-loaded passion flower leaf extract by nanoprecipitation to enhance its pharmacological activity. Results indicated that the extract exhibited low virulence, and high biocompatibility, and its antidepressant effect was similar to oral nortriptyline (Alves et al., 2020). Hypericin, an extract of *Hypericum perforatum*, is efficacious in improving mild and moderate depressive states. Waqar et al. (2023) evaluated the antidepressant activity of chrysin using nanoemulsion as a carrier for transnasal delivery. Tan et al. (2024) improved the poor water solubility, low bioavailability, and poor permeability of chrysin by using black phosphorus nanosheets as a carrier, significantly alleviating depressive symptoms. Besides, icariin primarily used for male reproduction, has shown potential therapeutic and preventive effects in the nervous system and can be used as an anti-inflammatory agent for depression, etc. (Jin et al., 2019; Luo et al., 2022). The emergence of natural antidepressants and the utilization of nano-drug delivery systems present encouraging prospects for the management of depression.

## 5 Application of nanocarriers in different routes of uptake

**FIGURE 2:**
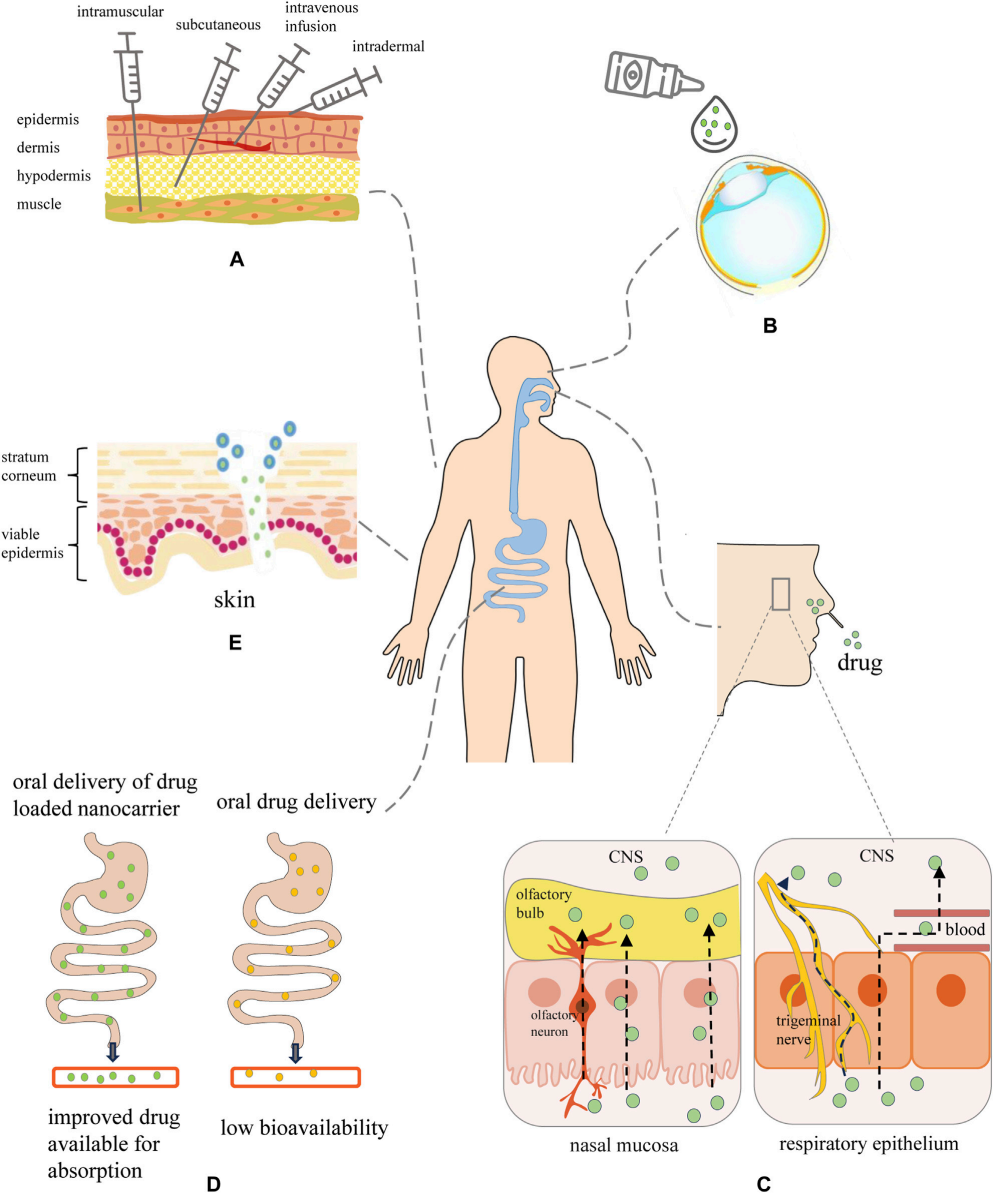
Graphical overview of the routes toward nanocarrier-mediated administration. **(A)** Injections: nanopreparations are administered via intramuscular, subcutaneous, intravenous, and intradermal. **(B)** Nano-based eye drop: enhanced ability to penetrate the ocular barrier and prolonged drug residence time at the ocular surface. **(C)** Intranasal transport route of therapeutic nanomaterials from the nasal cavity to the brain through nasal mucosa, trigeminal nerve and respiratory epithelium. Intranasal drugs may be transported to the olfactory bulb by paracellular or transcellular transport along olfactory neurons or across the nasal epithelium. Within the nasal respiratory epithelial region, the drug would travel via the trigeminal nerve to the CNS, or traverse the respiratory epithelium to access the bloodstream prior to entering the CNS. **(D)** Oral: nanocarrier-encapsulated drug protects the drug from destruction by digestive enzymes and improves drug absorption through the intestinal epithelium. **(E)** Transdermal: the interaction between nanocarriers and skin is influenced by regulating the lipophilicity, size, shape, and surface charge of the nanocarriers, which improves the skin penetration efficiency of the nanocarriers.

Oral, intranasal, and injection are the extensively studied routes for utilizing nanocarriers in depression treatment. There is an overview of the various methods for delivering nanocarriers (Figure 2).

### 5.1 Oral

Oral administration is the ideal route of administration for antidepressants because it is non-invasive, convenient, and low-cost. Nevertheless, oral medications undergo metabolism by the gastrointestinal wall or liver, which can diminish absorption rates and consequently reduce their effectiveness (Sato et al., 2023). Besides, the complex physiology of the BBB limits drug absorption. A variety of nanocarriers have been developed for oral antidepressants to improve the neuroavailability of drugs in the brain.

The curcumin extract (TUR) has been shown to have good antidepressant activity in various animal studies, and low oral solubility and malabsorption limit its clinical use. Sheng et al. designed an orally administered TUR nanoemulsion (TUR-NE) using PEG-400 and Tween 80 as surfactants to alleviate this problem (Sheng et al., 2023). The developed TUR-NE has a 5-fold increase in peak plasma concentration compared to the generic drug. Furthermore, in the model of Chronic Unpredictable Mild Stress (CUMS), tissue sections from the kidney and heart in the fluoxetine hydrochloride group were stained with mild lesions, whereas no lesions were evident in the TUR-NE group. The results showed that the nanoemulsions improve the water-solubility of the drug, which is more easily absorbed by the gastrointestinal tract and enhances the pharmacokinetics of the orally administered drug.

El-Helaly and Rashad (2024) established a polymeric micelle-based delivery platform to improve the oral bioavailability of mirtazapine. Pharmacokinetics showed that mirtazapine-loaded polymeric micelles (MTZ-PM) reached the maximum drug concentration faster than commercial mirtazapine tablets. This is because polymeric micelles improve the solubility and stability of the drug in the gastrointestinal tract and are more readily absorbed by the intestinal cells with the assistance of surfactants. They utilized the adsorption of Aerosil 200 to make the MTZ-PM liquid formulation into a dry powder, which was then mixed with a proportional surfactant and compressed into tablets. This provides the pharmaceutical industry with new ideas for making polymer micelles into oral tablets with high stability and bioavailability. The development of NDDS has undoubtedly opened up more possibilities for oral drug delivery for the treatment of depression.

### 5.2 Injection

Injectable administration delivers the drug directly into the circulation and avoids first-pass metabolism thus improving the efficacy of antidepressants. KA has been shown favorable antidepressant effect in treatment-resistant depression and is commonly delivered through intravenous infusion (Le Nedelec et al., 2018). Le Nedelec et al. (2018) compared plasma drug concentrations following the administration of KA through various routes in rats, including subcutaneous (SC), intramuscular (IM), intravenous infusion (IVI), and intravenous bolus (IVB) methods. Liquid chromatography-mass spectrometry measurements indicated that administration by the IBV route produced significantly elevated Cmax levels in comparison to alternative administration routes. Del Sant et al. (2020) monitored changes in blood pressure and heart rate in patients with treatment-resistant depression after subcutaneous injections of esketamine. It was found that there was minimal variation in blood pressure after repeated subcutaneous injections of esketamine and was effective in patients with comorbidities such as hypertension and diabetes mellitus. A randomized, double-blind trial designed by Loo et al. (2023) aimed to evaluate the antidepressant effectiveness and safety of repeated subcutaneous administrations of KA. This aligns with the findings of Del Sant et al.’s experimental study, suggesting that subcutaneous administration may offer a more straightforward and efficient approach to drug delivery.

### 5.3 Intranasal

Intranasal administration can be used for local, systemic, and even central nervous system administration (Keller et al., 2022). At the nasal cavity, drug molecules might bypass the BBB and be absorbed from the nasal mucosa into the brain via the trigeminal and olfactory pathways (Erdő et al., 2018). Compared to oral or intravenous administration, intranasal administration is non-invasive, fast-acting, has no first-pass metabolism and reduces side effects. Xu et al. (2020) designed a complex system loaded with Icariin to improve the antidepressant activity of Icariin and observed its antidepressant effect by nasal administration. Results suggested that the drug could be released continuously for 36 h, and fluorescently labeled icariin nanogel was detected in the brains of mice after 5 min of intranasal administration and reached the maximum fluorescence intensity within 30 min. In the classic CUMS animal model of depression, nasal administration shortened forced swimming and tail suspension times compared to oral administration. This suggests that nasal administration allows the drug to reach the brain quickly and accumulate, increasing the antidepressant activity of the drug (Xu et al., 2020). Intranasal administration has been approved primarily for treating localized systemic diseases, and its use in CNS disorders has attracted widespread interest. The clinical trials show the advantages of transnasal administration of esketamine in relieving depressive symptoms and preventing relapse (Daly et al., 2019; Popova et al., 2019; An et al., 2021).

## 6 Toxicity of nano-based antidepressants

In recent years, the application of nanoparticles has included biological imaging, targeted drug delivery, cancer vaccine delivery, mediated photothermal therapy, photodynamic therapy, etc. (Denkova et al., 2018; Zhao et al., 2022). In addition, nanomaterials have applications in brain tumors, epilepsy, Parkinson’s, Alzheimer’s, and other central system diseases (Islam et al., 2020). Numerous patents have been filed for nanoparticle drug delivery technologies for treating and diagnosing neurological disorders (Muheem et al., 2021).

As mentioned above, the nanocarriers can enter the body to achieve therapeutic effects by ingestion, inhalation, injection, and skin absorption. Oral administration might be the most commonly used route, and nanocarriers have shown an excellent future for oral drug delivery. However, studies have reported that the intestine is also an essential part of the immune system, maintaining the balance of intestinal flora and resisting oral pathogens. Although oral nanoformulation can improve the bioavailability of drugs, it may also cause an immune response (Ojer et al., 2015). Hort et al. (2021) encapsulated the drug within nanoemulsions (NE) and administered them to rats via oral and intraperitoneal injections of 200, 400, or 800 mg/kg of body weight. Results exhibited that oral ingestion of NE did not induce alterations in biochemical parameters in rats, whereas intraperitoneal injection at 800 mg/kg resulted in an inflammatory reaction. This finding indicates that the oral intake of nanoemulsions is safe, whereas elevated doses of parenteral administration may lead to adverse toxic responses (Hort et al., 2021). In addition, other studies have reported that nanoparticles can quickly accumulate in the brain after passing through the BBB, enter nerve cells, and produce neurotoxicity. Neurotoxic effects can directly affect the structure and function of the nervous system, thus affecting the function of the BBB (Teleanu et al., 2018). It is necessary to consider nanocarriers’ toxicity, metabolism, side effects, and other factors before delivering nanoformulations to the body through nano-drug delivery systems.

Toxicity assessment of nanoparticles is performed mainly *in vivo* and *in vitro*. *In vitro* techniques are commonly used to detect cytotoxicity, reactive oxygen species levels, immunotoxicity, carcinogenicity, hepatocytotoxicity, and genotoxicity by fluorescent probes, MTT, ELISA, and Tepan blue dye. The investigators simulated the performance of nanocarriers in the human body through animal models to ensure the safety of nanocarriers in clinical applications. The toxicity of nanoparticles is related to various factors such as route of administration, ingested dose, surface modification, particle size, shape, polydispersity coefficient, and others (Teleanu et al., 2018; Azarnezhad et al., 2020). Despite nanoparticles being extensively evaluated in laboratory and animal studies during the preclinical stage, their potential effects on the human body remain uncertain. To transfer nanoparticles from the laboratory to the clinic, it is necessary to master the interactions of nanoparticles with various tissue cells and blood components in the healthy or diseased state of the human body. This requires a complex and rigorous standard system to comprehensively evaluate nanoparticles from synthesis and characterization to drug delivery. Furthermore, the implementation of nanomedicine in clinical settings requires the consideration of intellectual property rights, governmental standardization, and market regulation, posing considerable challenges for both the pharmaceutical industry and regulatory bodies (Muheem et al., 2021). Ahmad et al. pointed out that nanotoxicology impacts the development and commercializing of nanocarriers, which requires the guidance of scientific teams and professional analysis (Ahmad et al., 2022).

## 7 Prospectives

NDDS provides essential opportunities for depression treatment. Currently used antidepressants have the disadvantages of high toxicity, poor permeability, and low bioavailability. NDDS encapsulates antidepressant drugs in nanoscale carriers, enabling precise release and targeting delivery *in vivo*, thereby improving therapeutic efficacy and patients’ quality of life. Although NDDS have shown excellent capability in treating depression, their clinical application still faces several challenges: 1) Toxicity studies are incomplete 2) Inadequate characterization techniques 3) Lack of standardized norms and regulations.

Much remains to be done to address the above issues. Firstly, The behavior of the nanocarriers *in vivo* such as circulation time, tissue distribution, and clearance rate needs to be determined. Also, the current commonly used toxicity evaluation methods need to be more accurate, and new toxicity evaluation strategies based on nanomaterials’ specificity in conjunction with nanotoxicology and neurology need to be designed (Szabat-Iriaka and Le Borgne, 2021). Secondly, there needs to be a transparent characterization scheme for the physicochemical properties of nanocarriers, which might be accountable for their letdown effect in late clinical trial stages. Interdepartmental collaboration between the National Cancer Institute (NCI), the National Institute of Standards and Technology (NIST), and the FDA established the Nanotechnology Characterization Laboratory (NCL) to improve the success of Phase II Human Trials. To adapt to the development of nanomedicine, NCL also needs to expand its popularity and communicate and collaborate with specialized pharmaceutical companies to understand the advances in nanomedicine preparation (Muheem et al., 2021). Finally, due to the specificity and complexity of nanotechnology, the traditional regulatory approach to drugs may not be able to fully cover nanomedicine, which will require multi-sectoral joint efforts to develop new regulations and regulatory measures (Mohapatra et al., 2024). The study of NDDS encompasses several academic disciplines, including but not limited to materials science, biology, pharmacology, etc. Strengthening interdisciplinary collaboration might promote the research and development of NDDS. Moreover, it is important to consistently exploring novel methodologies and technological domains, such as the integration of artificial intelligence and nanotechnology, as well as improving the design, material selection, and fabrication processes of nanocarriers.

Nanocarrier systems improve efficacy and reduce side effects by increasing the bioavailability of the drug, enabling targeted delivery and controlled release, thus sheding light on the novel solution through depression treatment may be developed.

## Figures and Tables

**Table:** 

**AAV**	adeno-associated viruses
**ABC**	ATP-binding cassette transporters
**AEDs**	anti-epileptic drugs
**BBB**	blood-brain barrier
**Cmax**	peak concentration
**CNS**	central nervous system
**CUMS**	chronic unpredictable mild stress
**EPR**	enhanced permeation and retention
**FST**	forced swim test
**FUS**	focused ultrasound
**HPLC**	high-performance liquid chromatography
**IGF-Ⅱ**	insulin-like growth factor II
**IM**	intramuscular
**IVB**	intravenous bolus
**IVI**	intravenous infusion
**KA**	ketamine
**MAOIs**	monoamine oxidase inhibitors
**NCL**	nanotechnology characterization laboratory
**NCI**	national cancer institute
**NDDS**	nanocarrier drug delivery system
**NE**	nanoemulsions
**NIST**	national institute of standards and technology
**NLC**	nanostructured lipid carriers
**OFT**	open field test
**PAMAM**	polyamide-amine
**PLGA**	polylactic acid-hydroxyacetic acid copolymer
**SC**	subcutaneous
**SNRIs**	selective norepinephrine and serotonin reuptake inhibitors
**SLN**	solid lipid nanoparticles
**SSRIs**	selective 5-hydroxytryptamine reuptake inhibitors
**TCAs**	tricyclic antidepressants
